# Restoration of a Critical Mandibular Bone Defect Using Human Alveolar Bone-Derived Stem Cells and Porous Nano-HA/Collagen/PLA Scaffold

**DOI:** 10.1155/2016/8741641

**Published:** 2016-03-28

**Authors:** Xing Wang, Helin Xing, Guilan Zhang, Xia Wu, Xuan Zou, Lin Feng, Dongsheng Wang, Meng Li, Jing Zhao, Jianwei Du, Yan Lv, Lingling E, Hongchen Liu

**Affiliations:** ^1^Institute of Stomatology, Chinese PLA General Hospital, Beijing 100853, China; ^2^Hospital of Stomatology, Shanxi Medical University, Taiyuan 030001, China; ^3^Department of Orthopedics, Chinese PLA General Hospital, Beijing 100853, China

## Abstract

Periodontal bone defects occur in a wide variety of clinical situations. Adult stem cell- and biomaterial-based bone tissue regeneration are a promising alternative to natural bone grafts. Recent evidence has demonstrated that two populations of adult bone marrow mesenchymal stromal cells (BMSCs) can be distinguished based on their embryonic origins. These BMSCs are not interchangeable, as bones preferentially heal using cells that share the same embryonic origin. However, the feasibility of tissue engineering using human craniofacial BMSCs was unclear. The goal of this study was to explore human craniofacial BMSC-based therapy for the treatment of localized mandibular defects using a standardized, minimally invasive procedure. The BMSCs' identity was confirmed. Scanning electron microscopy, a cell proliferation assay, and supernatant detection indicated that the nHAC/PLA provided a suitable environment for aBMSCs. Real-time PCR and electrochemiluminescence immunoassays demonstrated that osteogenic markers were upregulated by osteogenic preinduction. Moreover, in a rabbit critical-size mandibular bone defect model, total bone formation in the nHAC/PLA + aBMSCs group was significantly higher than in the nHAC/PLA group but significantly lower than in the nHAC/PLA + preinduced aBMSCs. These findings demonstrate that this engineered bone is a valid alternative for the correction of mandibular bone defects.

## 1. Introduction

Periodontal bone defects caused by periodontal disease, trauma, surgery, or tumor resection are very common and thus consume a large amount of medical resources annually. Autogenous bone (AB) grafting is the gold standard to reconstruct these defects, but sources of donor tissue are limited. Allografts have not been extensively applied in clinical practice due to the associated risks of antigenicity and cross-infection [1]. Synthetic materials have been studied extensively as potential bone substitutes, but, due to a lack of inherent osteogenic cells and osteoinductivity, limited formation of new bone occurs after osteoconduction is achieved [2].

As one approach to overcome these problems, biomaterial- and autologous stem cell-based tissue engineering has attracted much attention as a promising alternative to natural bone grafts. Among adult stem cells, mesenchymal stromal cells (MSCs) are favorite candidate seed cells for bone tissue regeneration because of their multipotency, immunomodulatory properties, and ability to release trophic factors [3, 4]. Although various adult tissue sources of MSCs, such as bone marrow, fat, muscle, dermis, and dental tissues, have been discovered, iliac bone marrow aspirates remain the principal source for bone regeneration [5, 6].

In preclinical and clinical studies, iliac-derived bone marrow mesenchymal stromal cells (BMSCs) have demonstrated promise for restoring bone defects and healing nonunion fractures [7, 8]. However, in clinical treatment, fear of pain and postoperative iliac complications has precluded the routine therapeutic use of BMSCs, particularly in dental patients who require alveolar bone augmentation. This issue suggests the need for the development of a more usable and minimally invasive procedure to isolate BMSCs [9, 10].

Recent evidence has suggested that at least two populations of adult skeletal progenitor cells can be distinguished based on their embryonic origins. Craniofacial bone arises from neural crest cells and ossifies via intramembranous ossification, whereas the appendicular skeleton arises from mesenchymal condensations of the mesoderm, which then undergoes perichondral ossification [11, 12]. Bridging craniofacial defects with grafts obtained from a craniofacial donor site is usually more successful than doing so with grafts from appendicular sites, indicating that skeletal site-specific differences affect graft integration [13, 14]. Furthermore, a selective recruitment mechanism through which adult skeletal defects heal, involving recruitment of progenitor cells of the same origin, has been demonstrated, indicating that BMSCs from the craniofacial and appendicular skeletons are not interchangeable [15]. The presence of two distinct populations of BMSCs in the adult might have clinical implications because if bones preferentially heal using cells that share the same embryonic origin, then reparative strategies may have to take this variable into account to be maximally effective. However, before the present investigation, the feasibility and effectiveness of tissue engineering using human craniofacial BMSCs to restore mandibular bone defects were unclear.

The goal of this study was to explore human craniofacial BMSC-based tissue engineering for the treatment of localized mandibular defects using a standardized, minimally invasive procedure. For this purpose, human alveolar BMSCs (aBMSCs) were isolated painlessly during conventional dental implant surgery, and the characteristics of the aBMSCs were analyzed in 2-dimensional cultures and on a porous nanohydroxyapatite/collagen/poly(L-lactide) (nHAC/PLA) scaffold. The osteogenic capability, biocompatibility, and biological safety of the constructs were also evaluated both* in vitro* and* in vivo*.

## 2. Materials and Methods

### 2.1. Isolation and Culture of aBMSCs

**Figure 1:**
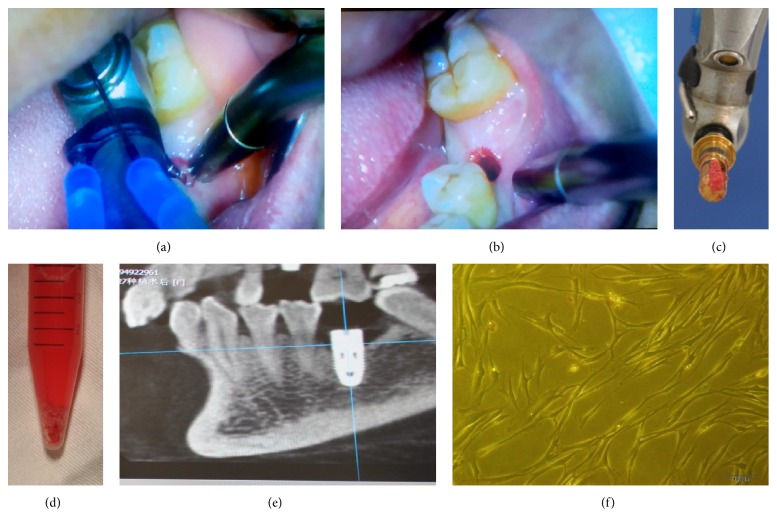
Clinical techniques for harvesting alveolar bone marrow. (a) The cancellous bone was drilled at 50 rpm until achieving the desired diameter and length for the implant site. (b) Before dental implant placement, bone marrow was harvested using a flapless, minimally invasive surgical procedure. (c, d) Bone marrow scraped from the pilot drill was immediately placed in sterile tubes and transported to the laboratory. (e) Confirmation of the initial stability of the dental implant by cone-beam CT. (f) Photomicrograph of aBMSCs 7–10 days following initial plating, revealing a fibroblastic, spindle shaped morphology similar to that of stem cells from other cell sources. Magnification: ×400.

Human alveolar bone marrow specimens were obtained from 52 patients during dental implant surgery following Chinese PLA General Hospital Review Board approval. In particular, after informed consent was obtained, we administered local anesthesia and removed the gingival flap and bone cortex at the implant sites using a flapless surgical procedure. Next, we gradually drilled into the cancellous bone at 50 rpm without irrigation until the desired diameter and length for the implant were obtained. Bone marrow scraped from the bone core that was harvested from the pilot drill was immediately placed in sterile tubes containing minimum essential alpha medium (*α*MEM; Gibco, Carlsbad, CA, USA) (Figure 1). Before dental implant placement, a 22.5-gauge needle connected to a heparinized syringe was inserted into the drill holes to obtain the marrow aspirate [16]. Both the collected debris and the aspirate were immediately transported to the laboratory for aBMSC isolation. The samples in *α*MEM medium were then centrifuged at 500 g for 5 minutes. The supernatant was removed, and the cell pellets were transferred to 25 cm^2^ culture flasks and resuspended in 5 mL of basic medium, consisting of *α*MEM supplemented with 15% fetal bovine serum (FBS; Gibco). The culture flasks were then incubated undisturbed, without medium changes, for 5 days in a 37°C humidified tissue culture incubator at 5% CO_2_. Once the cell density reached approximately 80% confluence, the cells were detached using trypsin/ethylenediaminetetraacetic acid (EDTA; Sigma, St. Louis, MO, USA) treatment and were passaged or used for subsequent analysis. Not all specimens could be included in every experiment because of the surgical schedule and the number of cells needed for each assay. In each experiment, cells obtained from different subjects were stored for analysis at the same time to avoid technical differences between assays.

### 2.2. Flow Cytometric Analysis

aBMSCs at passage 3 were trypsinized, washed, and resuspended in phosphate-buffered saline (PBS; Gibco) at a concentration of 1 × 10^5^ cells/mL. The cells were subsequently immunolabeled with mouse monoclonal anti-human antibodies specific for the following: CD14, CD34, CD44, CD90, CD106, and HLA-DR (Abcam, Cambridge, MA, USA). Mouse isotype antibodies served as controls. The cells were then washed again with PBS and fixed in 2% paraformaldehyde, after which the immunolabeled cells were detected by flow cytometry (BD Biosciences, NJ, USA).

### 2.3. Immunofluorescence

aBMSCs at passage 3 were detached using solution of versene (EDTA/PBS) and were subcultured on 12-chamber slides for 24 hours. The samples were then fixed in 2% paraformaldehyde for 15 minutes, followed by incubation with anti-STRO-1 antibody (1 : 200; R&D Systems Inc., Minneapolis, MN, USA) for 3 hours. The cells were subsequently incubated with anti-mouse IgG TRITC (1 : 50; Santa Cruz Biotechnology, Inc., USA) for 1 hour and stained with 4′,6-diamidino-2-phenylindole (DAPI; 2 *μ*g/mL; Sigma).

### 2.4. Multilineage Differentiation

aBMSCs at passage 3 were cultured on six-well culture plates at a density of 2 × 10^5^ cells/cm^2^ in induction medium. For osteogenic differentiation, the osteogenic induction medium consisted of *α*MEM, 15% FBS, 10 nM dexamethasone, 100 mM glycerophosphate, and 50 *μ*g/mL ascorbic acid [17]. The medium was renewed two times each week. At day 21, the cells were stained with Alizarin red.

For adipogenic differentiation, the induction medium consisted of 200 *μ*M indomethacin, 1 *μ*M dexamethasone, 0.5 mM 3-isobutyl-1-methylxanthine, and 100 nM insulin. The medium was renewed two times each week. At day 21, the cells were fixed and stained with fresh Oil red O solution. As a control, the same batch of aBMSCs was cultured in *α*MEM containing 15% FBS and stained at day 21.

### 2.5. Biometric Preparation and Seeding of nHAC/PLA Scaffolds

The features of the nHAC/PLA porous scaffold (Allgens, Beijing, China) were similar to that of natural bone: a porosity of 70–90% and a pore size of 300–400 ± 150 *μ*m [18]. aBMSCs at passage 3 were seeded into nHAC/PLA materials that had been cut into 10 × 5 × 3 mm blocks. The nHAC/PLA + aBMSCs constructs were then incubated in basic medium in a 24-well plate for 24 hours at 37°C. After the aBMSCs had adhered to the nHAC/PLA, the constructs were cultured in basic medium or osteogenic induction medium for* in vitro* characterization and* in vivo* implantation.

### 2.6. MTS Assay

3-(4,5-Dimethylthiazol-2-yl)-5-(3-carboxymethoxyphenyl)-2-(4-sulfophenyl)-2H-tetrazolium, inner salt (MTS; Promega), was used as an indicator of cell viability. In preparation, aBMSCs at passage 3 were seeded onto nHAC/PLA discs in 96-well culture plates at density of 1.5 × 10^4^ cells/cm^2^. After culture for 24 hours, the cells were serum starved overnight in *α*MEM with 1% FBS and then cultured in basic medium (nHAC/PLA + aBMSCs group) or osteogenic induction medium (nHAC/PLA + preinduced aBMSCs group) for another 7 days. On days 1, 3, 5, and 7 during this period, 20 *μ*L of MTS was added, and the samples were incubated for 2 hours at 37°C. Subsequently, the absorbance of each well was measured at 490 nm using an ELx800 UV reader (Bio-Tek Instruments, Winooski, VT, USA).

### 2.7. Scanning Electron Microscopy (SEM)

aBMSCs were cultured on nHAC/PLA scaffolds in basic medium or osteogenic induction medium at 1 × 10^5^ cells/cm^2^ for 7 days, after which the samples were washed with PBS and fixed in 2.5% glutaraldehyde. The samples were then coated with several nanometer-thick layers of gold. The adhesion and morphology of the aBMSCs on the surface of the nHAC/PLA composite were observed using a variable-pressure scanning electron microscope (S-3400N, Hitachi, Japan) with beam energies of 6–25 kV.

### 2.8. Osteogenic Gene

aBMSCs on nHAC/PLA scaffolds were cultured in osteogenic induction medium or basic medium for 7 days. Total RNA was subsequently extracted using TRIzol (Invitrogen, Grand Island, NY, USA), and first-strand complementary DNA (cDNA) was synthesized using a cDNA synthesis kit (Promega, Madison, WI, USA). Quantitative real-time PCR was then performed using human osteocalcin (OCN), Runx2, Osterix, and *β*-actin primers and Fast SYBR Green MasterMix in a StepOnePlus*™* Real-Time PCR System (Applied Biosystems, Carlsbad, CA, USA). The following primer sets were used: Runx2, GenBank Accession number 860, F5′-CGGAATGCCTCTGCTGTTATGAA-3′, R5′-AGGATTTGTGAAGACGGTTATGG-3′; Osterix, number 121340, F5′-CTCCTCCTGCGACTGCCCTAAT-3′, R5′-AGGTG CGAAGCCTTG CCATACA-3′; OCN, number 632, F5′-GGAGGGCAGCGAGGTAGTGAAG-3′, R5′-GATGTGGTCA GCCAACTCGTCA-3′; actin, number 60, F5′-TGCCCATCTA CGAGGGGTATG-3′, R5′-TCCTTAAT GTCACGCACGATTTC-3′. The expressions of the genes were normalized to the internal control b-actin mRNA levels. Data were analyzed using the comparison Ct (2^−ΔΔCt^) method. The specificity of single-target amplification by each primer pair was confirmed by melting curve analysis.

### 2.9. Alkaline Phosphatase (ALP), OCN, Calcium, and Phosphonium Content Assays

aBMSCs on nHAC/PLA scaffolds were cultured for 21 days in basic medium or osteogenic induction medium at 1.5 × 10^4^/cm^2^ cells per graft. The supernatants were collected at 3 time points: days 7, 14, and 21. The ALP activity in the medium was assayed using Roche Diagnostics ALP kits on the cobas e602 platform (Roche Diagnostics, Mannheim, Germany), and calcium content and phosphonium content were assayed using Roche Diagnostics Ca/P kits. OCN activity was assayed using N-MID osteocalcin kits, based on electrochemiluminescence immunoassay techniques. All data were measured and analyzed on the cobas 8000 platform (Roche Diagnostics).

### 2.10. Genetic Stability Assay

Reverse transcription PCR was performed to examine the gene expression of tumor suppressors and protooncogenes. For this purpose, RNA was isolated from aBMSCs cultured on flasks at passages 1 and 2 and from aBMSCs cultured on nHAC/PLA scaffolds in basic medium or osteogenic induction medium at passage 3. The primer sequences were as follows: p53, F5′-CCTCACCATCATCACA CTGG-3′, R5′-TTATGGCGGGAGGTAGACTG-3′; c-myc, F5′-CTCCTGGCAAAAGGTCAG AG-3′, R5′-GGCCTTTTCATTGTTTTCCA-3′; ARF, F5′-TGGGTCCCAGTCTGCA GTTA-3′, R5′-CTGCCCATCATCATGACCT-3′; GAPDH, F5′-ACAGTCAGCC GCATCTTCTT-3′, R5′-ACGACCAAAT CCGTTGACTC-3′.

At the end of culture, aBMSCs from three different patient samples at passage 4 were subjected to karyotyping analysis. These aBMSCs were cultured on nHAC/PLA scaffolds in osteogenic induction medium, followed by treatment with 60 ng/mL colcemid (Sigma) and harvesting with 0.25% trypsin/EDTA. The cells were then collected by centrifugation, and karyotyping analysis was performed using G-banding techniques in a clinical laboratory at the Chinese PLA General Hospital.

### 2.11. Bone Regeneration in the Mandibular Defect Model

The bone-forming capacity of 20 different aBMSCs populations was evaluated qualitatively in a New Zealand white rabbit segmental critical-size mandibular defect model [18, 19]. For this purpose, 30 female rabbits were housed in the laboratory animal center at the Chinese PLA General Hospital. The rabbits had weights of 2.50–3.00 kg. All surgical procedures and care followed Chinese PLA General Hospital Review Board approval. The rabbits were first intravenously anesthetized with 2% pentobarbital sodium (30 mg/kg). The hair on the right of the rabbit mandible was then depilated. Under aseptic conditions, a 10 mm incision was made along the upper edge of the rabbit mandible, and the right buccal was exposed. A segmental defect (10 × 5 × 3 mm) was prepared in the alveolar bone of all rabbits using a surgical oscillating saw and sterile saline irrigation. nHAC/PLA blocks with/without 5 × 10^6^ aBMSCs were implanted in the segmental defect, and the rabbits were assigned to five groups: mandibular defects were treated with nHAC/PLA, nHAC/PLA + aBMSCs, or nHAC/PLA + preinduced aBMSCs (preinduced in osteogenic induction medium for 7 days) or with AB from the iliac bone as a positive control or untreated defect as a negative control.

After eight weeks, the samples were surgically removed and fixed. Goldner's trichrome staining was used to examine bone regeneration at the defects. All samples were analyzed by morphometric analysis using the Leica-Q Win 3.2 image analysis system. Five sequential sections per implant were selected, and the type of tissue (mature bone-like or osteoid-like) was identified by an independent observer. The extent of newly formed bone was indicated by the percentage of the newly formed bone areas within a section. An average value was then calculated for each implant, and data were averaged across all implants within each group.

### 2.12. Assessment of Bone Regeneration by Micro-CT

The bone volume and trabecular microarchitecture were monitored using a Quantum FX micro-CT Imaging System (Caliper, USA) at 70 kV and 114 mA. A total of 512 binary images were obtained with a wide field of view (FOV), with scanning at 36 mm and a 4.5 *μ*m voxel size resolution. The thresholds were set at 1300, which was adequate to separate bone and nHAC using discrimination analysis. The calculated bone volume density (bone volume/total volume) in this area is presented as the percentage regeneration of the defect.

### 2.13. Statistical Analysis

All data are presented as the mean ± standard deviation. Statistical analyses of the results were performed using SPSS 16.0 software. Student's *t*-test was used to study significant differences in the MTS, PCR, SEM, ALP, OCN, and calcium and phosphonium content results. One-way ANOVA and an unpaired *t*-test were used to study significant differences in the bone volume density and the percentage of bone formation area between study groups. The significance threshold was set at *p* < 0.05.

## 3. Results

### 3.1. Isolation and Characterization of aBMSCs

We collected alveolar bone marrow samples from 52 patients undergoing dental implant surgery. Every dental implant was initially stable (final torque > 35 Ncm), and no complications after bone marrow collection were reported. Postoperative 1- to 3-month cone-beam CT results revealed closely aligned dental implants and bone (Figure 1(e)).

**Figure 2:**
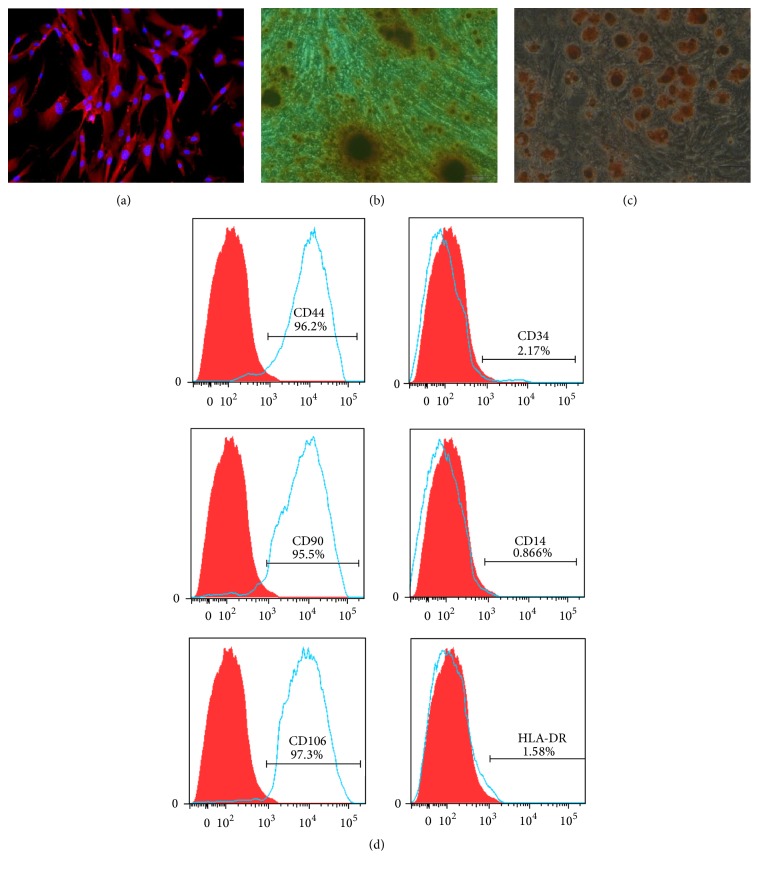
Characterization of human aBMSCs. (a) The aBMSCs expressed STRO-1, a mesenchymal stromal cell marker. Positive staining of the cells with Alizarin red (b) or Oil red O (c) demonstrated the osteogenic and adipogenic differentiation potential of the aBMSCs. Magnification: ×400. (d) The aBMSCs were positive for MSC markers, including CD44, CD90, and CD106, and negative for hematopoietic and endothelial markers, including CD14, CD34, and HLA-DR.

The aBMSCs in 37 samples adhered to the culture surface and proliferated* in vitro*. The isolated aBMSCs formed single-cell-derived colonies, and most of the cells retained their fibroblastic spindle shape (Figure 1(f)). The aBMSCs were expanded in culture, followed by characterization of cell surface markers by flow cytometry and immunofluorescence. Immunofluorescence analysis demonstrated that the cells were STRO-1 positive (Figure 2(a)). To evaluate the* in vitro* differentiation potential of the aBMSCs, the cells were induced to differentiate along osteogenic and adipogenic lineages under specific culture conditions, as revealed by Alizarin red (Figure 2(b)) and Oil red O (Figure 2(c)) staining. Flow cytometry revealed that the cultured cells were homogenously positive for CD44, CD90, and CD106 and negative for hematopoietic and endothelial cell surface markers, including CD14, CD34, and HLA-DR (Figure 2(d)).

### 3.2. Biocompatibility between aBMSCs and nHAC/PLA

**Figure 3:**
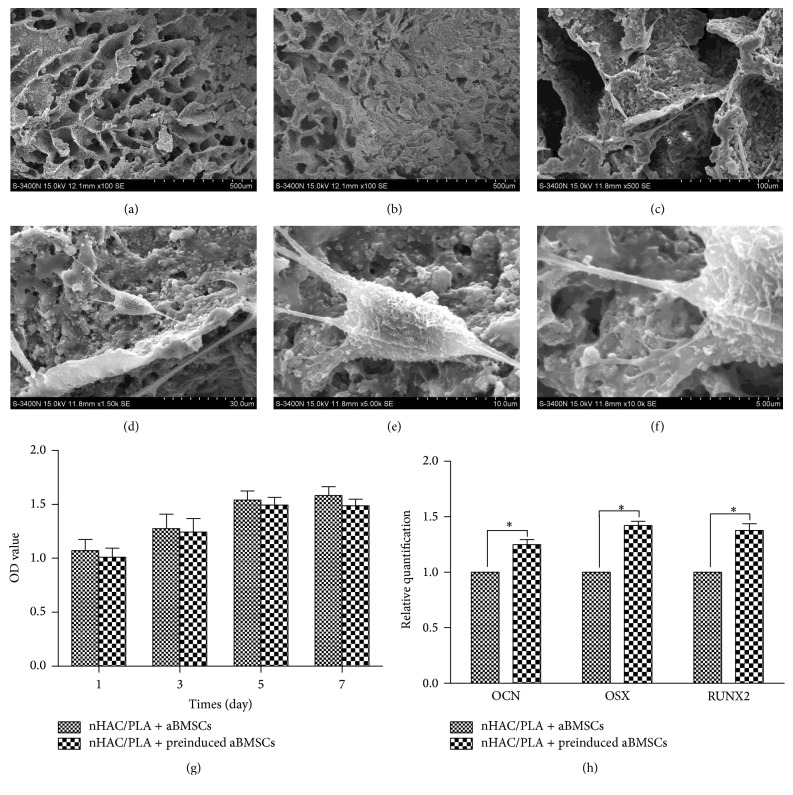
Proliferation and osteogenic differentiation potential of aBMSCs on nHAC/PLA. (a) SEM analysis showed that nHAC/PLA was similar to natural bone in terms of microstructure. (b) After 3 days of culture, the aBMSCs adhered to, extended, and connected with each other and produced ECM on the nHAC/PLA surface. The aBMSCs (c–f) were spindle, triangle, or cube shaped, with developed cytoplasmic extensions attached to the scaffold. (g) The MTS results demonstrated that the aBMSCs were proliferative on days 1, 3, 5, and 7 after seeding onto nHAC/PLA (*n* = 6, mean ± SD). (h) Real-time PCR revealed that osteogenic induction for 7 days promoted aBMSC expression of OCN, Runx2, and Osterix (*n* = 6, mean ± SD).  ^*∗*^Compared with nHAC/PLA + aBMSCs. Differences are significant at *p* < 0.05. Magnification: (a, b) ×100 SE; (c) ×500 SE; (d) ×1.50 k SE; (e) ×5.00 k SE; (f) ×10.0 k SE.

The adherence and morphology of aBMSCs on nHAC/PLA were observed by SEM, which revealed that the nHAC/PLA blocks without cells exhibited a hierarchical microstructure (Figure 3(a)). After 3 days of culture, the aBMSCs adhered to, extended, and connected with each other and produced extracellular matrix (ECM) on the nHAC/PLA surface (Figure 3(b)). The aBMSCs were spindle, triangle, or cube shaped, with developed cytoplasmic extensions attached to the scaffold (Figures 3(c)–3(f)).

The proliferation of aBMSCs on nHAC/PLA was evaluated using the MTS method on days 1, 3, 5, and 7 (Figure 3(g)). Throughout the 7 days of culture, the cell numbers increased, indicating that the nHAC/PLA had no negative effect on proliferation. Compared with cells cultured in basic medium (nHAC/PLA + aBMSCs group), the number of aBMSCs on nHAC/PLA decreased in the nHAC/PLA + preinduced aBMSCs group, but the difference was not significant (*p* > 0.05).

### 3.3. Osteogenic Capability of aBMSCs on nHAC/PLA

To confirm their osteogenic potential, aBMSCs on nHAC/PLA were cultured in basic medium or osteogenic induction medium for 7 days, followed by the evaluation of osteogenesis-related genes by quantitative real-time PCR. The PCR results demonstrated that osteogenic inductive treatment promoted expression of the osteogenesis-related genes OCN, Runx2, and Osterix in the BMSCs (Figure 3(h)).

**Figure 4:**
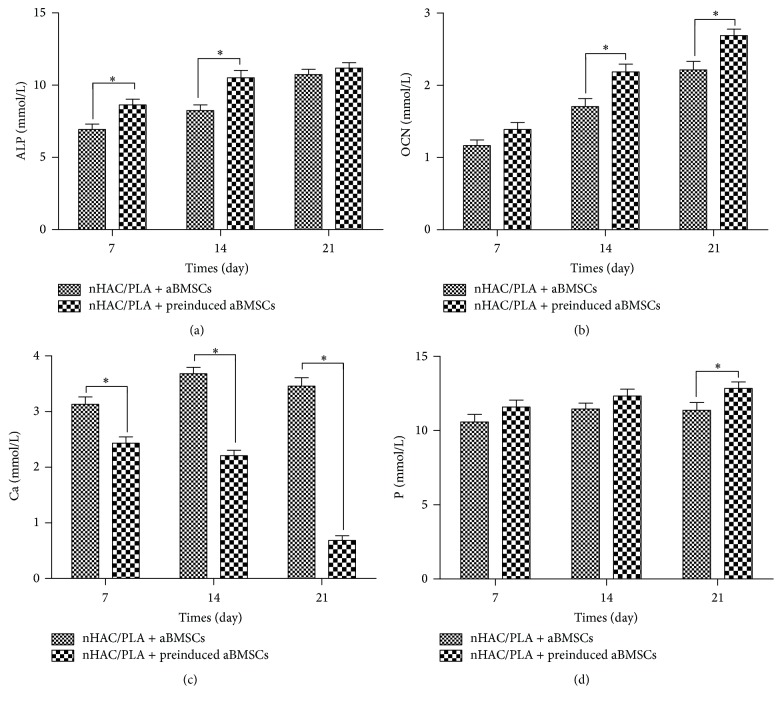
Effect of osteogenic induction on the ALP activity (a), OCN content (b), calcium content (c), and phosphonium content (d) of aBMSCs cultured on nHAC/PLA (*n* = 6, mean ± SD).  ^*∗*^Compared with nHAC/PLA + aBMSCs. Differences are significant at *p* < 0.05.

To investigate the osteogenic capability of aBMSCs on nHAC/PLA, the ALP activity, calcium and phosphonium content, and OCN content in the culture supernatant were assayed at days 7, 14, and 21. The aBMSCs and nHAC/PLA constructs were cultured in basic medium or osteogenic induction medium for this purpose. During the 28 days of culture, the ALP activity and OCN and phosphonium content in both groups increased with culture time, but the calcium content decreased. However, the ALP activity and OCN and phosphonium content were significantly higher in the nHAC/PLA + preinduced aBMSCs group than in nHAC/PLA + aBMSCs group at day 21 (Figure 4). Although the calcium content in both groups declined, the content in the nHAC/PLA + preinduced aBMSCs group was always significantly lower.

### 3.4. Genetic Stability Assessment

**Figure 5:**
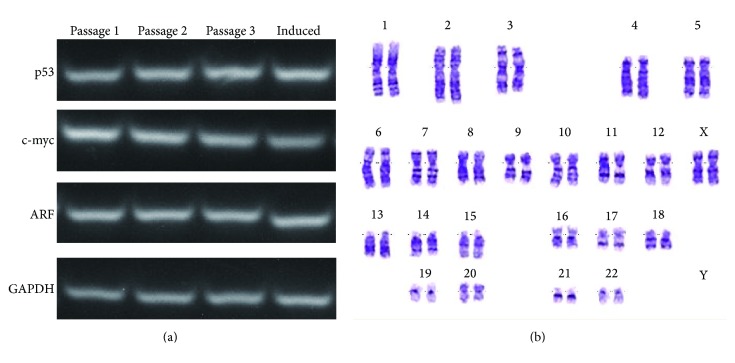
Genetic stability evaluation. (a) The expression levels of p53, ARF, and c-myc in aBMSCs were unchanged over time and under osteogenic induction. (The term “induced” denotes aBMSCs at passage 3 that were cultured on nHAC/PLA scaffolds in osteogenic induction medium.) (b) aBMSCs at passage 4 that were cultured on nHAC/PLA in osteogenic induction medium exhibited a normal karyotype.

To evaluate transformational alterations in gene expression, the levels of the tumor suppressor genes p53 and ARF and the protooncogene c-myc were measured (*n* = 6). However, no significant changes were observed over time or upon osteogenic induction (Figure 5(a)).

After culture on nHAC/PLA scaffolds in osteogenic induction medium for 21 days, aBMSCs at passage 4 exhibited a normal diploid karyotype (*n* = 6). Chromosome structural abnormalities such as inversion, deletion, translocation, and rings were not observed by karyotyping analysis of G-banding (Figure 5(b)).

### 3.5. Bone Regeneration in a Mandibular Defect Model

**Figure 6:**
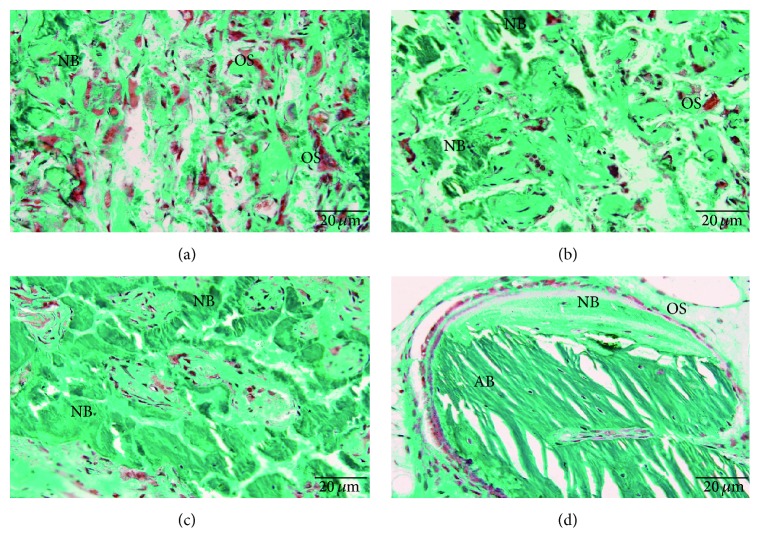
Bone regeneration in a rabbit critical-size mandibular defect model stained with Goldner's trichrome. (a) The defects in the nHAC/PLA group were filled with abundant red-stained osteoid tissue and some green-stained newly formed bone. (b) The defects in the nHAC/PLA + aBMSCs group were filled with more newly formed trabeculae. (c) The defects in the nHAC/PLA + preinduced aBMSCs group were filled with a large amount of mature thickened bone. (d) The AB group exhibited areas of newly formed bone, osteoid tissue, osteoblastic seams, and residual bone graft. Magnification: ×400. NB: new mature bone; AB: autogenous bone; OS: osteoid tissue.

In the critical-size mandibular defect rabbit model, the mandible was harvested for histological analyses at 8 weeks. In the nHAC/PLA and the nHAC/PLA + aBMSCs groups, the mandibular defects were filled with abundant red-stained osteoid tissue and some green-stained newly formed bone (Figures 6(a) and 6(b)), and many osteoclasts were observed in the newly formed osteoid tissue. Meanwhile, the defects in the nHAC/PLA + preinduced aBMSCs group were filled with a large amount of mature thickened bone (Figure 6(c)), and osteoblasts lined the surface of the newly formed bone. In the AB group, there were areas of autogenous grafted bone as well as areas of new bone regeneration (Figure 6(d)). In different maturation stages, the autologous bone and newly formed bone exhibited different trabecular arrangements. Little bone regeneration was observed in the untreated defect group, which served as the negative control.

A total of 68 slides from the different groups were quantified by morphological analysis. The extent of newly formed mature bone and osteoid tissue was indicated by the percentage of total bone formation area within the section; this percentage was averaged across all slides within each group. The percentages of the osteoid tissue formation area in the nHAC/PLA, nHAC/PLA + aBMSCs, nHAC/PLA + preinduced aBMSCs, and AB groups were 23.5 ± 3.3, 15.6 ± 1.2, 7.9 ± 1.9, and 9.2 ± 1.1, respectively. The percentages of mature bone formation area in the nHAC/PLA, nHAC/PLA + aBMSCs, nHAC/PLA + preinduced aBMSCs, and AB groups were 3.2 ± 1.2, 19.2 ± 1.3, 41.2 ± 2.4, and 43.2 ± 2.7, respectively. The percentages of total bone formation area in the nHAC/PLA, nHAC/PLA + aBMSCs, nHAC/PLA + preinduced aBMSCs, and AB groups were 26.7 ± 4.5, 34.8 ± 2.5, 49.1 ± 4.3, and 52.2 ± 3.8, respectively.

**Figure 7:**
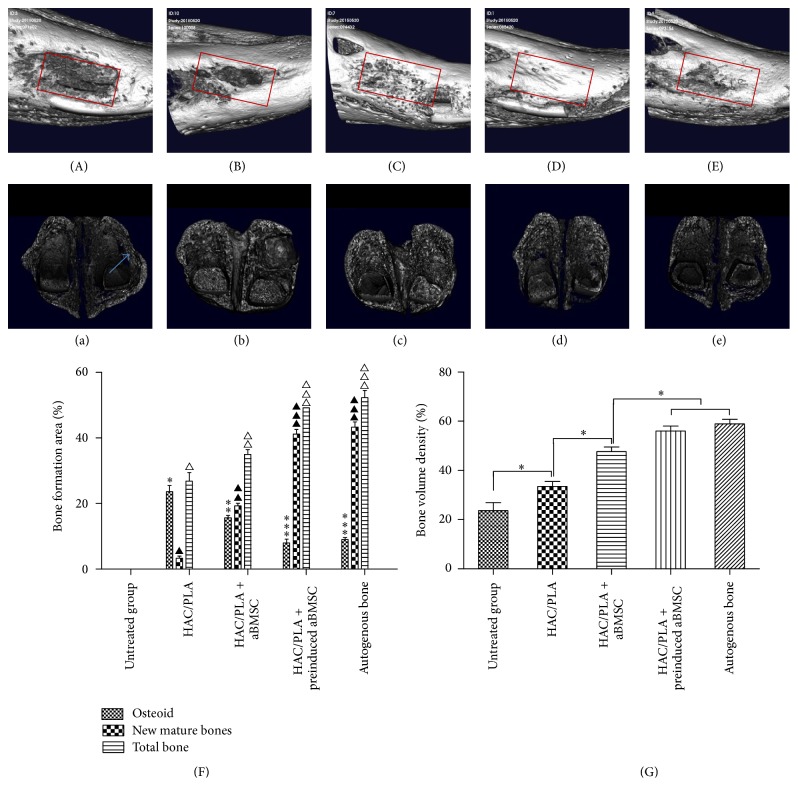
Micro-CT images of the mandibular defects at 8 weeks after implantation. (A, a) The critical-size bone defect in the untreated group was unfilled. (B, b) For defects filled with an nHAC/PLA scaffold, new bone formation in the open scaffold pores, with incomplete healing on the surface of the defect, was observed. (C, c) The defect surface was healed with a thin cortical shell bridge in the nHAC/PLA + aBMSCs group. (D, d) The defect surface was completely healed with a thick cortical shell bridge in the nHAC/PLA + preinduced aBMSCs group. (E, e) In the AB group, the defect healed well, and the iliac graft block and mandibular bone could not be distinguished. (The red boxes represent the defect area in the mandible, and the blue arrow indicates an incomplete cortical shell.) (F) The percentages of osteoid tissue formation, mature bone formation, and total bone formation were determined by histomorphometric measurements. Differences are significant at *p* < 0.05. Groups with the same symbol (*∗*, ▲, ▵) were not significantly different. (G) The bone volume density in micro-CT is presented as the percentage regeneration of the defect. Differences are significant at *p* < 0.05.

Histomorphometric measurements revealed significant differences in bone formation (Figure 7(F)). The nHAC/PLA group had the lowest percentage of total bone formation area of the five implanted groups and differed significantly from the other groups (*p* < 0.05). Total bone formation in the nHAC/PLA + aBMSCs group was significantly higher than in the nHAC/PLA group (*p* < 0.05) but significantly lower than in the nHAC/PLA + preinduced aBMSCs and the AB groups (*p* < 0.05). The maximal percentage of total bone formation area was observed in the AB group. However, there were no significant differences between the nHAC/PLA + preinduced aBMSCs and the AB groups (*p* > 0.05).

Regeneration of the mandibular defects was evaluated using micro-CT. In the 3-dimensional volume reconstruction of the images, the bone defect in the untreated defect group was unfilled (Figure 7(A)). For defects filled with an nHAC/PLA scaffold, new bone formation in the open scaffold pores, with incomplete healing of the defect, was observed (Figure 7(B)). For defects filled with nHAC/PLA + aBMSCs, the defect surface was healed with a thin cortical shell bridge (Figure 7(C)). For defects filled with nHAC/PLA + preinduced aBMSCs, the defect surface was also completely healed with a thick cortical shell bridge (Figure 7(D)). In the AB group, the defects healed well, and the iliac graft block and mandibular bone could not be distinguished (Figure 7(E)).

To quantify new bone formation, the bone volume density in the defect is presented as the percentage regeneration of the defect. The bone volume densities in the nHAC/PLA, nHAC/PLA + aBMSCs, nHAC/PLA + preinduced aBMSCs, and AB groups and the untreated defect group were 25.1 ± 4.5, 33.2 ± 3.4, 47.8 ± 4.2, 55.4 ± 3.9, and 58.5 ± 3.8, respectively (Figure 7(G)). The bone volume density in the nHAC/PLA + aBMSCs group was significantly higher than in the nHAC/PLA group (*p* < 0.05) but significantly lower than in the nHAC/PLA + preinduced aBMSCs and the AB groups (*p* < 0.05). There were no significant differences between the nHAC/PLA + preinduced aBMSCs and the AB groups (*p* > 0.05), indicating that new bone formation was significantly improved by the nHAC/PLA + preinduced aBMSCs.

## 4. Discussion

As a promising alternative approach for the treatment of bone defects, bone tissue engineering creates a bone grafting material with osteogenic, osteoinductive, and osteoconductive properties. These events are also essential for optimal bone healing when a combination of BMSCs and biomaterials is to be used to treat periodontal bone defects.* In vitro* studies of BMSCs often involve cells derived from the tibia of animals, including mice and rats. When human skeletal progenitor cells are studied, most bone grafting procedures performed for craniofacial applications use cells derived from the mesodermal lineage (e.g., the fibula, iliac crest, and ribs). Recently, several scholars have described the isolation of BMSCs from the marrow of maxillofacial bones and demonstrated that these cells share the basic characteristics of MSCs [10, 16]. In the same individuals, maxillofacial-derived BMSCs were found to have greater osteogenic potentials than iliac crest-derived BMSCs [20]. In previous studies, we also found that osteogenic differentiation capacity of alveolar BMSCs was higher than that of femoral BMSCs in the middle-aged and old group [21]. These reports indicate the BMSCs from maxillofacial bones with some characteristics that may be beneficial for treating maxillofacial bone defects. Nevertheless, in most published studies, alveolar BMSCs have not been used in critical-size mandibular bone defect model.

In this study, we describe a convenient and minimally invasive process for craniofacial BMSC isolation and further demonstrate that a combination of aBMSCs after osteogenic induction and nHAC/PLA could effectively restore mandibular defects. It would be an advantage to use aBMSCs via this process, thereby reducing donor-site morbidity and residual pain related to bone harvesting from, for example, the iliac crest.

At the histological level, defect repair in the craniofacial skeleton is indistinguishable from appendicular bone repair: both defect sites are vascularized following trauma, both become populated by osteoblast progenitor cells, and both undergo bony matrix remodeling within a similar time frame. However, cellular and molecular analyses belie this histological equivalency. In particular, Hox expression status as well as embryonic origin has an influence on the fate of skeletal progenitor cells in the regenerative context. Leucht et al. found that, initially, mandibular skeletal progenitor cells are Hox negative but that they adopt a Hoxa11-positive profile when transplanted into a tibial defect. Conversely, tibial skeletal progenitor cells are Hox positive and maintain this Hox status even when transplanted into a Hox-negative mandibular defect [15]. This mismatch between the Hox gene expression statuses of host and donor cells is correlated with a disruption in bone regeneration, such that the grafted cells fail to differentiate into osteoblasts [22]. When iliac crest-derived BMSCs are placed in a mandibular defect, these mesoderm-derived progenitor cells differentiate into chondrocytes [23]. Conversely, craniofacial bone-derived BMSCs can integrate into and contribute to bony regeneration in a mandibular defect.

In the present study, we isolated and characterized aBMSCs, which fulfill the criteria of the International Society for Cellular Therapy, including fibroblast-like morphology, expression of surface markers, and a capacity for multilineage differentiation [24]. Although the maintenance of stemness is difficult to achieve in* ex vivo* culture, this profile suggests that cells collected from alveolar bone resemble immature mesenchymal cells. We also determined that the aBMSCs were STRO-1 positive. STRO-1, which recognizes only clonogenic and highly osteogenic progenitors, is expressed by stromal elements in the bone marrow [25]. Additionally, we evaluated the osteogenic potential of the aBMSCs. Runx2 and Osterix are essential transcription factors for osteoblastic differentiation and skeletal morphogenesis, and OCN is a major noncollagenous protein specific to bone and the most recently identified osteogenic expression marker.

The cellular state under 2D and 3D cell culture conditions may vary considerably [26]. Accordingly, the proliferation and osteogenic capabilities of the aBMSCs on the 3D porous nHAC/PLA scaffold were further evaluated. The nHAC/PLA scaffold was formed from new mineralized collagen consisting of a combination of collagen fibrils and PLA attached to nanohydroxyapatite by a self-assembly method. The microstructure of this composite was a mineralized collagen fiber bundle, similar to the hierarchical structure of natural bone [27]. We have previously demonstrated that this composite has good biocompatibility and osteoconductivity, suggesting its potential for hard tissue repair [18]. The results of the current study further confirmed that nHAC/PLA provides a suitable environment for aBMSC adhesion, proliferation, and differentiation. The upregulation of both osteogenic gene expression and protein expression suggests that the osteogenic potential of aBMSCs on nHAC/PLA was activated by the osteogenic induction medium.

In this study, a localized mandibular defect model was used to characterize the osteogenic differentiation of the nHAC/PLA + aBMSCs construct* in vivo*. Micro-CT and histomorphometric analyses further confirmed the capability of this method to repair bone defects. In the rabbit mandible, a defect diameter greater than 5 mm has been reported to be a critical size that prevents spontaneous healing [19]. In the present study, after 8 weeks of implantation, histological analysis demonstrated that the untreated group exhibited no bone formation and that the nHAC/PLA group had abundant engineered osteoid tissue and some newly formed bone. These results indicated that a critical-size defect was successfully established and that nHAC/PLA can be used as a potential scaffold for mandibular bone regeneration. Furthermore, the nHAC/PLA + aBMSCs group had significantly greater bone formation than the nHAC/PLA group did. These results once again confirmed that the structure and composition of the nHAC/PLA had good biocompatibility and promoted cell proliferation and osteogenic differentiation. The ECM and other factors in the bone defect environment played a key role in this osteogenic differentiation of the aBMSCs. Specifically, for defects filled with nHAC/PLA + preinduced aBMSCs, a large amount of mature thickened bone was observed in the morphometric analysis, and the defect surface exhibited complete healing, with a thick cortical shell bridge in CT images. These results demonstrated that the nHAC/PLA + aBMSCs preinduced in osteogenic induction medium did indeed exhibit enhanced initial bone formation* in vivo*. These results also indicated that factors from defects are insufficient to stimulate aBMSCs to undergo osteogenic differentiation. To achieve improved therapeutic effects, regulating the degree of aBMSCs' osteogenic differentiation is desirable. The selected inducer may include osteoblasts that have undergone osteogenic preinduction* in vitro*. Osteoblast-secreted factors can promote the proliferation and osteogenic differentiation of BMSCs via the VEGF/heme-oxygenase-1 pathway [28]. To verify that the tissue-engineered bone generated from nHAC/PLA + preinduced aBMSCs is a valid alternative for the reconstruction of mandibular bone defects, a positive-control, gold-standard fresh autogenous iliac bone graft was also tested in this study. Quantitative histomorphometric and micro-CT analyses demonstrated that the tissue-engineered bone was similar to that derived from the gold-standard method.

The effects of the nHAC/PLA + preinduced aBMSCs method on bone defect repair are similar to those of the autologous bone method, but with less trauma. Therefore, the method presented here needs to be considered carefully for clinical applications. Cone-beam CT also confirmed that the method does not affect the osseointegration of dental implants. From a purely technical perspective, aBMSC collection offers advantages over collection from other sites because the cells are passed through the mucous membrane, without damaging the skin, which avoids the need for general anesthesia and effectively reduces patient discomfort. Moreover, the cells can be easily obtained during routine dental surgery, such as dental implant surgery, wisdom tooth extraction, and tooth crown-lengthening measures. The selection of surgical methods depends on the surgeon's familiarity with the anatomic region, but most maxillofacial surgeons and dentists feel confident about extracting bone marrow from alveolar bones.

It is noteworthy that although complete absence of teratoma formation was observed in this study, it is only a speculation whether some aBMSCs still survive* in vivo* or are killed in xenogeneic hosts. Several studies have suggested that the stem cells could suppress immune responses through immune-privileged, immunosuppressive, or tolerance-inducing methods [29, 30]. Others have suggested that the functional improvements after implantation of xenogeneic stem cells are only caused by paracrine effects rather than by engraftment [31]. Thus, further studies about immune mechanisms will be necessary to correctly interpret the results of animal models and for future translation into clinical practice.

Another safety consideration for tissue engineering strategies is genetic stability in* ex vivo* cell culture. In the present study, aBMSCs were expanded, passaged, and osteogenically induced. Following long-term cell culture, the results of karyotyping, tumor suppressor, and protooncogene analyses were unchanged. These results are in agreement with those of Poloni et al., who observed that human adipocytes dedifferentiated into endothelial cells and did not undergo transformational changes [32]. Our results thus demonstrated that genetic instability does not occur among aBMSCs cultured on nHAC/PLA in osteogenic induction medium.

## 5. Conclusion

The key parameter of bone reconstruction in bone tissue engineering is the selection of favorable seed cells, biomaterials, and an osteogenic inducer; changes in any of these three factors will affect osteogenesis. The present study overcame the difficulties of isolating human craniofacial BMSCs and demonstrated the bone regeneration potential of an innovative seed cell type, namely, aBMSCs, without viral gene delivery. These findings also demonstrated that this method of applying nHAC/PLA + aBMSCs with osteogenic preinduction is a valid alternative for the correction of mandibular bone defects. This study is an important step in the clinical application of human craniofacial BMSCs, although additional studies will be necessary to determine the effects of immunity on human autologous aBMSCs after preinduction* in vitro*.
